# Short-Term Exposure to High Sucrose Levels near Weaning Has a Similar Long-Lasting Effect on Hypertension as a Long-Term Exposure in Rats

**DOI:** 10.3390/nu10060728

**Published:** 2018-06-06

**Authors:** Mariana Villegas-Romero, Vicente Castrejón-Téllez, Israel Pérez-Torres, María Esther Rubio-Ruiz, Elizabeth Carreón-Torres, Eulises Díaz-Díaz, Leonardo del Valle-Mondragón, Verónica Guarner-Lans

**Affiliations:** 1 Department of Physiology, Instituto Nacional de Cardiología “Ignacio Chávez”, Juan Badiano 1, Sección XVI, Tlalpan, Mexico City 14080, Mexico; mvromero21@gmail.com (M.V.-R.); vcastrejn@yahoo.com.mx (V.C.-T.); esther_rubio_ruiz@yahoo.com (M.E.R.-R.); 2 Department of Pathology, Instituto Nacional de Cardiología “Ignacio Chávez”, Juan Badiano 1, Sección XVI, Tlalpan, Mexico City 14080, Mexico; pertorisr@yahoo.com.mx; 3 Department of Molecular Biology, Instituto Nacional de Cardiología “Ignacio Chávez”, Juan Badiano 1, Sección XVI, Tlalpan, Mexico City 14080, Mexico; qfbelizabethcm@yahoo.es; 4 Department of Reproductive Biology, Instituto Nacional de Ciencias Médicas y Nutrición “Salvador Zubirán”, Vasco de Quiroga 15, Sección XVI, Tlalpan, Mexico City 14000, Mexico; eulisesd@yahoo.com; 5 Department of Pharmacology, Instituto Nacional de Cardiología “Ignacio Chávez”, Juan Badiano 1, Sección XVI, Tlalpan, Mexico City 14080, Mexico; leonardodvm65@hotmail.com

**Keywords:** critical window, hypertension, sucrose, endothelial nitric oxide synthase, oxidative stress, fatty acids

## Abstract

Adverse conditions during early developmental stages permanently modify the metabolic function of organisms through epigenetic changes. Exposure to high sugar diets during gestation and/or lactation affects susceptibility to metabolic syndrome or hypertension in adulthood. The effect of a high sugar diet for shorter time lapses remains unclear. Here we studied the effect of short-term sucrose ingestion near weaning (postnatal days 12 and 28) (STS) and its effect after long-term ingestion, for a period of seven months (LTS) in rats. Rats receiving sucrose for seven months develop metabolic syndrome (MS). The mechanisms underlying hypertension in this model and those that underlie the effects of short-term exposure have not been studied. We explore NO and endothelin-1 concentration, endothelial nitric oxide synthase (eNOS) expression, fatty acid participation and the involvement of oxidative stress (OS) after LTS and STS. Blood pressure increased to similar levels in adult rats that received sucrose during short- and long-term glucose exposure. The endothelin-1 concentration increased only in LTS rats. eNOS and SOD2 expression determined by Western blot and total antioxidant capacity were diminished in both groups. Saturated fatty acids and arachidonic acid were only decreased in LTS rats. In conclusion, a high-sugar diet during STS increases the hypertension predisposition in adulthood to as high a level as LTS, and the mechanisms involved have similarities (participation of OS and eNOS and SOD expression) and differences (fatty acids and arachidonic acid only participate in LTS and an elevated level of endothelin-1 was only found in LTS) in both conditions. Changes in the diet during short exposure times in early developmental stages have long-lasting effects in determining hypertension susceptibility.

## 1. Introduction

Early programming is the process by which adverse conditions during the early stages of development, such as malnutrition, produce long-term consequences for the structure and function of the organism [1]. If diet is altered during gestation and lactation, when epigenetic regulations are fixed [2,3], the descendants will show permanent physiological and biochemical changes, that may help survival and growth at early stages but may have undesirable long-term costs; such as increasing predisposition to diseases (obesity, metabolic syndrome, hypertension) [4,5,6]. 

In many cases, hypertension may be determined by conditions undergone during the intrauterine period, infancy and childhood [7]. Changes in lifestyle habits such as reducing maternal smoking, increasing breastfeeding, reducing salt consumption in infancy and preventing childhood obesity have been suggested as possible interventions to prevent hypertension during adulthood [7]. Most models of fetal programming of hypertension used relatively long-term perturbations that included gestation and lactation. These alterations also produced a low birth weight in the offspring [8,9,10,11]. The decreased growth, in utero, might alter renal development causing hypertension. Feeding rats with a high sucrose diet before and during pregnancy and during lactation and the first days after weaning produced a higher incidence of hypertension when the offspring reached adulthood [12]. A high salt diet before and during pregnancy as well as during lactation and the early weaning period also increased the incidence of hypertension [13]. A high salt diet for a shorter lapse of time, only during gestation, increased the risk of the female offspring rats having elevated blood pressure (BP) as adults [14].

However, these studies tested alterations in the diet during long periods of time. Alterations during shorter periods of time, known as critical windows, have not been tested. A critical period around weaning (from postnatal day 12 to 28) has been described in rats, during which there are important developmental changes in the pancreas, as well as changes in glucose and insulin concentrations [15]. In previous studies, we found that hyperglycemia and hyperinsulinemia modify adult vascular responses [16]. Metabolic syndrome (MS) rats that have hyperglycemia and hyperinsulinemia and hypertriglyceridemia have modified vasoreactivity [16]. We have also previously described changes in aortic contractility during this critical window lasting 16 days around weaning where plasmatic glucose and insulin concentrations vary [17].

It has been described that the energetic metabolism in the mitochondria leads to the production of metabolites that participate in the establishment of epigenetic cues, and which could participate in early programming [18,19,20]. Since insulin and glucose modify vascular contractility in the critical window near weaning, and carbohydrate metabolism participates in the establishment of epigenetic cues, here we studied whether variations in sucrose ingestion during the critical window might determine hypertension susceptibility during adulthood.

The aims of the present paper also include: (1) to study the mechanisms underlying the effect of short-term sucrose exposure (STS) coinciding with the critical window, on the development of hypertension in the adult; (2) to study the mechanisms underlying hypertension after long-term ingestion of sucrose (seven months) (LTS), in which rats develop metabolic syndrome (MS), since the mechanisms underlying hypertension in sucrose-induced MS have not been reported; and (3) to compare the mechanisms underlying hypertension in both models. The mechanisms might be the same for STS and LTS, since the ingestion of sucrose in rats developing MS includes the critical window near weaning or they could differ since the lapse of time during which sucrose is ingested is longer. 

We report that the susceptibility to develop hypertension in the adult is increased when the diet is modified during the critical window around weaning by the ingestion of high sucrose. We study whether hypertension is due to endothelin-1 or nitric oxide (NO) production alterations in STS and LTS rats. We analyze three possible mechanisms underlying hypertension when the animals reach adulthood in both models: the first is through the effect of insulin, which inhibits endothelial nitric oxide synthase (eNOS) [21]; the second is through an elevation of free fatty acids, mainly oleic acid, which might diminish the activity of eNOS; and the third is through oxidative stress (OS), since uncoupled eNOS and other enzymes generate reactive oxygen species that react with NO, diminishing its bioavailability and therefore vessel relaxation [22]. We found that OS and the altered expression of eNOS and SOD2 participate in STS and LTS and that fatty acids and arachidonic acid only participate in LTS. An elevated level of endothelin-1 was only found in LTS rats.

## 2. Materials and Methods

### 2.1. Animals and Experimental Groups

The animal experiments were approved by the Laboratory Animal Care Committee of the Instituto Nacional de Cardiología “Ignacio Chávez” in Mexico and were conducted in compliance with our institution’s ethical guidelines for animal research.

Wistar inbred rats with no predisposition to cardiometabolic diseases from the animal facility of our institution were used. A total of 15 litters of rats were divided into three experimental groups including 5 litters each: (a) Control rats that received tap water throughout the experiment; (b) Rats that were given 30% sucrose in drinking water from postnatal day 12 to 7 months of age (LTS), who developed metabolic syndrome [23]; and (c) Rats that received 30% sucrose in drinking water during the possible postnatal critical window for increased hypertension susceptibility (postnatal days 12 to 28) (STS) and were then given tap water until 7 months of age. Litters consisted of 8 male pups and the mother. Therefore, a total of 40 male pups and 5 mothers per experimental group were used. Although small differences might be expected between female and male pups during the weaning stage, since sexual hormone production is low, hormones might induce differences in the adult stage. Only male animals were studied, since the MS model has been better characterized in males. Differences between male and female organisms should be the aim of another study. In all groups, weaning took place on postnatal day 21. Therefore, the critical window included the time when pups begin to drank water but still suckled and a period during which they only drank water. 

All animals were fed Purina 5001 rat chow (Richmond, IN, USA) ad libitum, and were kept under controlled temperature and a 12:12-h light-dark cycle. Pups were placed in metabolic cages at the end of the critical window to determine sucrose ingestion. Water intake (mL/day) was 34.25 ± 0.98 in controls and 20.75 ± 0.85 in pups ingesting sucrose, the difference being statistically significant (*p* < 0.05). The kilocalorie (kcal) equivalents ingested with the sucrose in the water was 24.9 ± 1.03. Food intake (g/day) was 15.17 ± 0.96 in controls and 8.25 ± 0.58 in sucrose-fed rats and was statistically different (*p* < 0.05). The kcal equivalents ingested with the food were also significantly different (*p* < 0.05), being 23.74 ± 1.5 in controls and 12.91 ± 0.91 in sucrose-fed rats. The total kcal equivalents ingested with the water and food were 23.75 ± 1.5 in controls and 37.81 ± 1.5 in sucrose-fed rats, showing a statistically significant difference (*p* < 0.05). Therefore, sucrose-fed rats ingested more kcal per day. Systolic arterial BP was measured in conscious adult animals using the tail cuff method; the cuff was connected to a pneumatic pulse transducer (Narco bio-systems Inc., Healthdyne Co., Austin, TX, USA) and a programmed electrosphyngomanometer. The mean of five independent determinations was calculated.

After overnight fasting (12 h), the animals were killed by decapitation and the blood was collected. The serum was separated by centrifugation at 600 *g* for 15 min at room temperature and stored at −70 °C until needed. The thoracic aortas were dissected and cleaned from the surrounding tissue.

### 2.2. Thoracic Aorta Homogenization

A sample from the thoracic aorta was taken for homogenization using a lysis buffer (25 mM 4-(2-hydroxyethyl)-1-piperazineethanesulfonic acid (HEPES), pH = 7.5; 100 mM NaCl, 10% Glycerol, 1% Triton-X100, 7 mg/mL sodium deoxycholate) supplemented with a mixture of protease inhibitors (1 mM phenylmethanesulfonyl fluoride (PMSF), 10 µg/mL pepstatin A, 10 µg/mL leupeptin and 10 µg/mL aprotinin) (Sigma Chemical Co., St. Louis, MO, USA). Tissue was homogenized in liquid nitrogen first, and then it was mixed with lysis buffer at 4 °C. The thoracic aorta homogenate was incubated 30 min, at 4 °C in nutator and centrifuged at 14,000 rpm for 10 min at 4 °C. The supernatant was separated and kept at −70 °C until required. The protein concentration was determined by the Bradford method (Protein assay, Bio-Rad laboratories Inc., Hercules, CA, USA) [24]. 

### 2.3. Biochemical Measurements

#### 2.3.1. Glucose, Insulin and HOMA-Index

Serum insulin was determined using a commercial radioimmunoassay (RIA) specific for rats (Linco Research, Inc., St. Charles, MO, USA); its sensitivity was 0.1 ng/mL and the intra- and inter-assay coefficients of variation were 5 and 10%, respectively. The glucose concentration was assayed using an enzymatic SERA-PAK^®^ Plus from Bayer Corporation (Bayer Corporation, Sées, France). The homeostasis model assessment of insulin resistance (HOMA-IR) was used as the physiological index of insulin resistance. The HOMA-IR was calculated from the fasting glucose and insulin concentrations using the following formula:

(insulin (µU/mL) × glucose (in mmol/L)/22.5) [25].

#### 2.3.2. Lipidic Profile

Total cholesterol (TC) and plasma triglyceride concentrations were measured in serum using commercial enzymatic assays (RANDOX Laboratories, Crumlin, UK). The high-density lipoprotein (HDL) cholesterol content was determined in the bottom fraction obtained after the ultracentrifugation of the plasma at density of 1.063 g/mL for 2.5 h at 100,000 rpm (Beckman optima TLX) [25,26]. The non-HDL-C is defined as the difference between the values of TC and HDL-C and includes low density lipoprotein cholesterol (LDLC), intermediate density lipoproteins (IDL), and very-low-density lipoproteins (VLDL). Recently non-HDL-C has become a commonly-used marker for a blood lipid pattern associated with an increased risk of heart disease. Fatty acids (FA) and non-esterified fatty acids were extracted and identified by gas liquid chromatography. Briefly, 100 g of protein from plasma were used in the presence of 100 g of nonadecanoic acid as the internal standard and 2 mL of chloroform–methanol (2:1, *v*/*v*) with 0.002% butyl hydroxytoluene (BHT), as described by Folch [27]. The total FA was trans-esterified to their FA methyl esters by heating them at 90 °C for 2 h with methanol, 2% in concentrated H_2_SO_4_ and 0.002% BHT. The total FA methyl esters were separated and identified by gas chromatography-flame ionization detector (FID) in a Carlo Erba Fratovap 2300 chromatograph equipped with a capillary column packed with the stationary phase—HP-FFAP (description: 30 m length × 0.320 mm diameter × 0.25 µm film)—and fitted with a flame ionization detector at 210 °C, with helium as the carrier gas at a flow rate of1.2 mL/min.

#### 2.3.3. Lipoperoxidation (LPO) 

LPO, a marker of damage by free radicals, was measured using a standard method [25]. One mg of protein from the thoracic aorta homogenate was used. A total of 50 µL CH3-OH with 4% BHT plus phosphate buffer pH 7.4 was added to 100 µg of aortic homogenate. The mixture was shaken vigorously in a vortex for 5 s and then incubated in a water bath at 37 °C for 30 min. A total of 1.5 mL of 0.8 M thiobarbituric acid was added and the sample was incubated in a water bath at boiling temperature for 1 h. After this time and to stop the reaction, the samples were placed on ice; 1 mL 5% KCl was added to each sample as well as 4 mL n-butanol, then they were shaken in a vortex for 30 s and centrifuged at 4000 rpm at room temperature for 2 min. The n-butanol phase was then extracted, and the absorbance was measured at 532 nm. The calibration curve was obtained using tetraethoxypropane as the standard [28].

#### 2.3.4. Total Antioxidant Capacity

A total of 100 µL of thoracic aorta homogenate was suspended in 1.5 mL of a reaction mixture of the three following solutions, in a relation of 10:1:1 *v*/*v*: (a) 300 mM acetate buffer pH 3.6; (b) 20 mM hexahydrate of ferric chloride; and (c) 10 mM of 2,4,6-Tris-2- pyridil-s-triazine dissolved in 40 mM chlorhydric acid. The mixture was shaken vigorously in a vortex for 5 s. It was then incubated at 37 °C for 15 min in the dark. The absorbance was measured at 593 nm. The calibration curve was obtained using Trolox [29].

### 2.4. Endothelial Nitric Oxide Synthase and SOD1 and 2 Immunoblotting

A total of 50 μg of aorta homogenate were mixed with 4× loading buffer (20% glycerol, 4% SDS, 0.02% bromophenol blue, 0.2% 2-mercaptoethanol, 125 mM Tris, pH 6.8). The mixture was heated at 100 °C for 5 min. The proteins were separated by sodium dodecyl sulfate polyacrylamide gel electrophoresis (SDS-PAGE), 8% bis-acrilamide-laemmli gel for endothelial nitric oxide synthase (eNOS), or, 15% bis-acrilamide-laemmli gel for superoxide dismutase (SOD) 1 or 2; and transferred to a 0.22 μm polyvinylidene difluoride (PVDF) membrane. Blots were blocked for 1 h at room temperature using Tris saline buffer plus 0.01% Tween (TBS-T) and 5% non-fat dehydrated milk. Afterwards, membranes were incubated overnight with a 1:1000 dilution, at 4 °C with primary antibodies, polyclonal rabbit anti-eNOS (sc-376751), mouse anti-SOD1 (sc-271014) and goat anti-SOD2 (sc-18503) (all from Santa Cruz Biotechnology, Santa Cruz, CA, USA). Then, the membranes were rinsed three times with TBS-T buffer and incubated for 3 h at room temperature with horseradish peroxidase conjugated secondary antibodies, dilution 1:10,000 (Santa Cruz Biotechnology). All blots were incubated with glyceraldehyde-3-phosphate dehydrogenase (GAPDH) antibody (sc-365062) (Santa Cruz Biotechnology, Santa Cruz, CA, USA) as a control. Protein was detected by a chemiluminescence assay (Clarity Western ECL Substrate, Bio-Rad Laboratories, Inc., Hercules, CA, USA). The chemiluminescence emitted in this process was detected in X-ray films (AGFA, Ortho CP-GU, Agfa HealthCare NV, Mortsel, Belgium). Images from each film were acquired with a GS-800 densitometer (including Quantity One software from Bio-Rad Laboratories, Inc., Hercules, CA, USA). The values of each band density are expressed as arbitrary units (AU).

### 2.5. Determination of NO

The seric concentration of NO was evaluated by UV-visible spectrophotometry according to the method of Tenorio [30]. A total of 100 µL of serum was deproteinized with cold methanol and centrifuged to 16,000 *g* for 15 min at 10 °C (Prism R, LabNet International, Inc. Palo Alto, CA, USA). A Sep-Pak Classic C-18 cartridge (Waters, Milford, MA, USA) was conditioned with phosphate buffer 100 mM pH 7.0 (pH-Meter HI-208, Hanna Instruments, Urbana, IL, USA) and used to filter the supernatant. The filtered solution was then treated with vanadium chloride (III) 0.8% in 1 M phosphoric acid and sulfanilamide 2% (*w*/*v*) in phosphoric acid 5% (*v*/*v*). Once the solution was homogenized, a solution of N-(1-naphthyl) ethylenediamine dihydrochloride 0.2% (*w*/*v*) in H_2_O was added. The reaction was incubated for 45 min at room temperature and finally 3 mL of water was added. The samples were spectrophotometrically analyzed at 572 and 587 nm (Evolution 220, Thermo Scientific, Urbana, IL, USA). The difference between the measurements (ΔAbs = 572–587 nm) was used to calculate the concentration of NO compared with a standard curve (0 to 200 pmol/mL).

### 2.6. Determination of Endothelin-1

Endothelin-1 was quantified in serum by high pressure liquid chromatography (HPLC), according to the method of Kumarathasan [31]. The sample was deproteinized in a 1:1.5 ratio with a cold mixture of methanol-1N HCl (40:1). It was vigorously homogenized (Vortex Mixer, Fisher Scientific, Urbana, IL, USA) for 1 min and centrifuged (Prism R Microcentrifuge, LabNet International, Inc. Palo Alto, CA, USA) at 16,000 *g* for 10 min at 10 °C. The supernatant was filtered with Millex-GV filters with hydrophilic Durapore PVDF membrane with 0.22 micrometer and 4 mm diameter pore with male Luer-Slip connection (Millipore, Billerica, MA, USA) and subsequently diluted in a 1:10 ratio with 0.1 M NaOH. The resulting mixture was separated by means of a translucent white PVC column of 5 mL capacity (12 mm ID × 100 mm), packed with Sephadex G-25 (GE Healthcare Life Sciences, Issaquah, WA, USA) in 0.1 N HCl. It was then filtered with a SepPak Classic C-18 solid phase extraction cartridge (55 μm, 70 Å, capacity: 100 mg/1 mL) (Waters Corporation, Milford, MA, USA), and finally with a 0.22 μm nitrocellulose membrane filter (Corning Incorporated, Corning, NY, USA) and directly analyzed. For this purpose, the equipment “Acquity HPLC System” coupled to a fluorescence detector (Waters Corporation, Milford, MA, USA), equipped with an Acquity column UPLC Peptide Separation Technology BEH300 C18 (1.7 μm, 21 × 50 mm) (Waters Corporation, Milford, MA, USA) was used. The separation was carried out by passing a gradient from 0 to 50% of phase B for 30 min at a flow of 0.2 mL/min (phase A: 0.02% trifluoroacetic acid in water, phase B: 0.018% trifluoroacetic acid in acetonitrile), with detection at 360 nm of excitation and 460 nm of emission at 10 °C. The concentration of endothelin-1 was compared with that in a standard curve (0 to 20 pmol/mL).

### 2.7. Statistical Analysis

Results are expressed as mean ± standard errors of the mean (SEM). For multiple comparisons, we applied one-way analysis of variance (ANOVA) followed by the post-hoc Tukey test. The Sigma Stat program (Jandel Scientific, San Rafael, CA, USA) was used. Differences were considered statistically significant when *p* < 0.05.

## 3. Results

### 3.1. Changes in Body Weight, Abdominal Fat, Glucose, Insulin, HOMA-IR

The values of these variables in control animals that did not receive sucrose during development are shown in Table 1 and correspond to previously reported values for adult rats. Animal that received 30% sucrose in the drinking water during a short period, corresponding to the possible critical window, and returned to tap water until six months of age (STS) showed similar values to those of control animals. Experimental adult LTS animals that received sucrose during and after the STS had a body weight similar to control animals but the abdominal fat was significantly increased. They had hypertriglyceridemia, hyperinsulinemia and HOMA-IR, as previously reported [23].

### 3.2. Changes in Blood Pressure, NO and Endothelin-1 Levels and eNOs Expression

**Figure 1:**
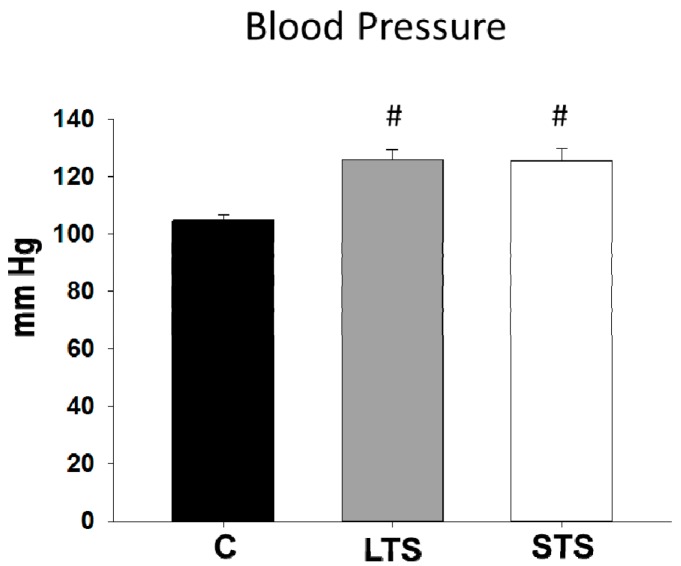
The effects of long-term (LTS) and short-term (STS) administration of sucrose on systolic blood pressure. C-control, MS-metabolic syndrome rats receiving sucrose for six months including the possible critical window, SP-rats receiving sucrose during the 16 days of the possible critical window for hypertension development around weaning and then receiving 5.5 months of tap water. Data represent mean ± SEM, # *p* < 0.01 after one-way ANOVA, *n* = 10 determinations from 10 different animals of each group.

Control rats showed normal levels of BP. Rats receiving sucrose during and after the critical window (LTS; MS model) showed arterial hypertension. Surprisingly, rats receiving 30% sucrose in the drinking water only during the STS and then returned to drinking tap water for six months had increased blood pressure reaching almost the same levels as LTS rats. (Table 1, Figure 1).

**Figure 2:**
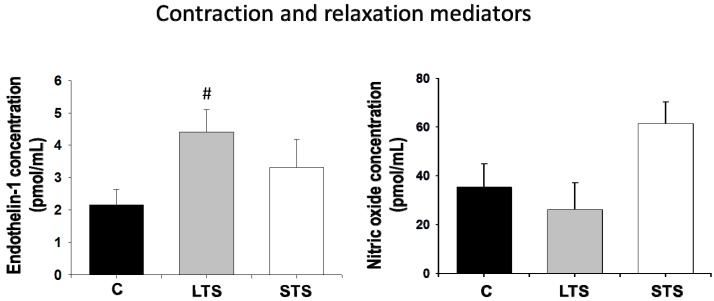
The effects of long-term (LTS) and short-term (STS) administration of sucrose on endothelin-1 and NO concentrations in plasma. Data represent mean ± SEM, # *p* < 0.05 after one-way ANOVA, *n* = 8 determinations from plasma from 8 different animals of each group.

**Figure 3:**
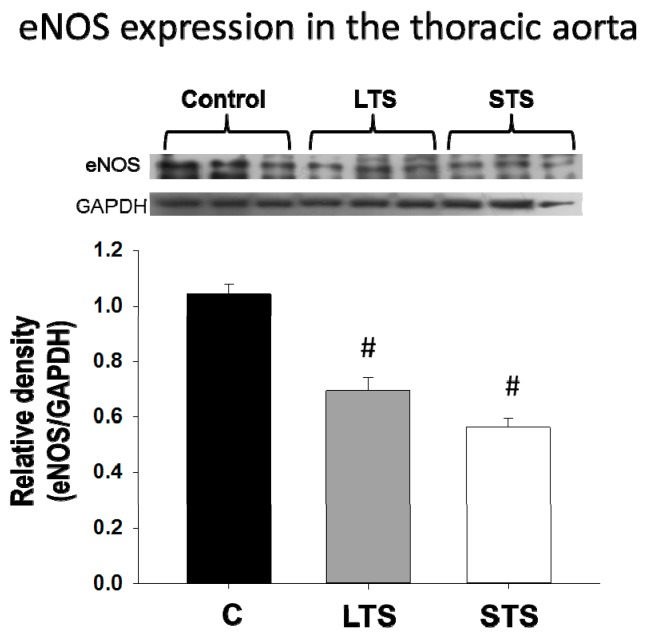
The effects of long-term (LTS) and short-term (STS) administration of sucrose on protein expression of eNOS. Data represent mean ± SEM, # *p* < 0.05 after one-way ANOVA, *n* = 8 lanes in Western blots in which homogenates of aortas from 8 different rats from each group were run. A representative Western blot analysis is shown above the graph.

Endothelin-1 concentration was increased in LTS rats but not in STS rats (Figure 2). There were no significant changes in NO in LTS rats or STS rats. eNOS protein expression decreased in both LTS rats and STS rats (Figure 3). 

### 3.3. Lipidic Profile and Free Fatty Acid Levels

Triglycerides were increased only in the LTS rats that developed MS. There were no significant changes in total cholesterol, HDL cholesterol and non-HDL cholesterol in any of the groups studied (Table 2). 

**Figure 4:**
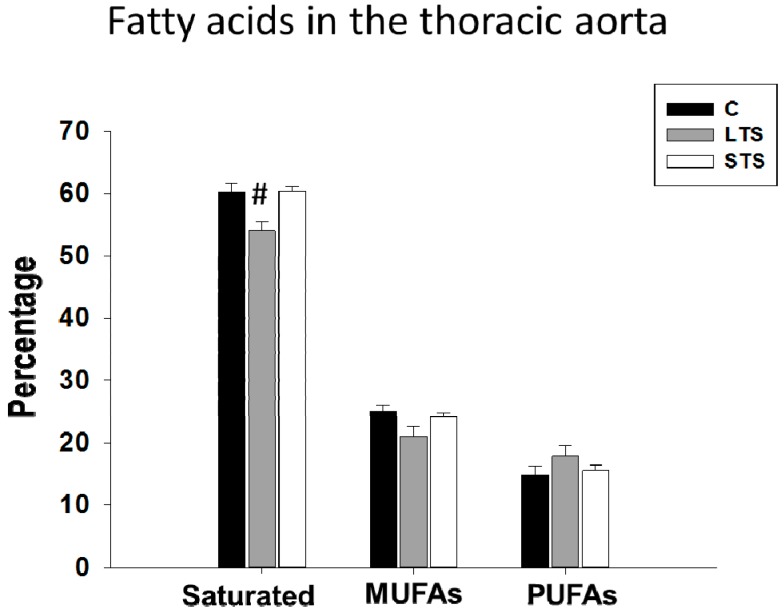
Effects of long-term (LTS) and short-term (STS) administration of sucrose on saturated fatty acids, monounsaturated fatty acids (MUFAs) and polyunsaturated fatty acids (PUFAs). Data represent mean ± SEM, # *p* < 0.05, *n* = 8 determinations from different rats in each group.

The saturated FA percentage was decreased in LTS rats but not in STS rats. Monounsaturated and polyunsaturated percentages of free FA were not modified in LTS or STS rats (Figure 4). Stearic and arachidonic acids were decreased in the LTS rats and linoleic acid was increased. In the STS rats, there were no changes in fatty acids when compared to control rats; the oleic acid levels remained unchanged in the three groups analyzed (Table 3). 

### 3.4. LPO, Total Antioxidant Capacity, SOD1, and SOD2 Expression

**Figure 5:**
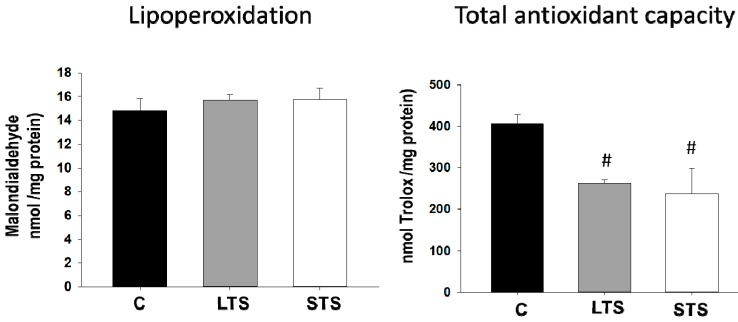
The effects of long-term (LTS) and short-term (STS) administration of sucrose on oxidative stress markers: lipoperoxidation (LPO) and total antioxidant capacity. Data represent mean ± SEM, # *p* < 0.05, *n* = 8 determinations from different rats in each group.

**Figure 6:**
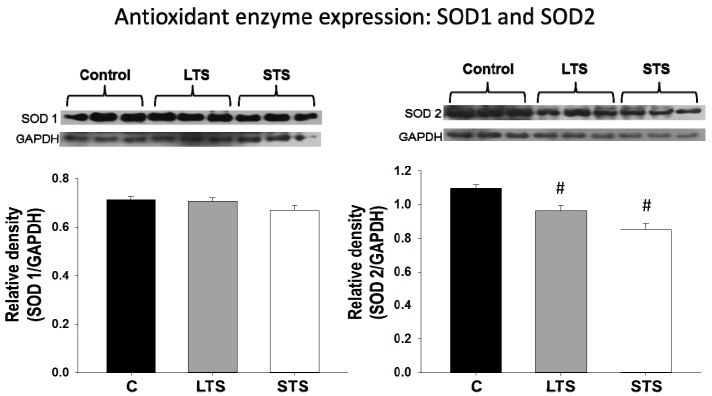
The effects of long-term (LTS) and short-term (STS) administration of sucrose on protein expression of the antioxidant enzymes SOD1 and SOD2. Data represent mean ± SEM, # *p* < 0.05, *n* = 8 lanes in Western blots in which homogenates of aortas from 8 different rats from each group were run. A representative Western blot analysis is shown above the graph.

The LPO remained unchanged in the LTS and STS rats, while the total antioxidant capacity was decreased in both experimental groups (Figure 5). The expression of SOD1 did not show significant changes with the treatments, while the expression of SOD2 was significantly decreased in the LTS and STS rat aortas (Figure 6).

## 4. Discussion

Treating high BP in middle age reduces the occurrence of cardiovascular disease; however, even well-controlled hypertensive adults still have reduced survival compared with normotensive subjects. Therefore, preventing hypertension from early life is important. There is increasing evidence that adult BP, may in some cases be determined by conditions established during the intrauterine period, infancy and childhood; this is also true for other cardiovascular diseases [8,32,33,34,35,36,37]. There is a potential relationship between childhood BP levels and adult hypertension [38]. Therefore, the study of the mechanisms that increase our predisposition to developing hypertension induced by changes in diet during early development are important. The exploration of the differences found with mechanisms produced by long-term alterations in diet that induce high BP is also important. 

Rodent models using high-fat and high-carbohydrate diets have been widely used to study human induced obesity, diabetes and their cardiovascular complications, rendering important advances in the knowledge of these diseases [39]. However, the reliability of results in rodent models is being questioned by some authors due to species-specific differences at every level of glucose regulation, from gene/protein expression, cellular signaling, tissues and organs, to the whole-organism level. Variations have been reported when comparing data obtained employing human cells, tissues, organs, and populations and when studying rodent models [40]. 

The sucrose concentration given to the rats in the present paper corresponds to the concentration found in many sodas [41]. This is the first report on the mechanisms underlying hypertension in STS and LTS models.

Regarding the effects of altered diets during the early stages of life and their effect in adults, the effect of long-term exposure to adverse diets—such as a diet containing high sucrose during gestation and lactation—on the development of hypertension has previously been studied. Contreras and coworkers [13] showed that giving female rats a high salt diet during pregnancy and lactation and for 10 days after weaning will produce a lasting increase in BP in the adult offspring. A high carbohydrate diet has also been tested during the gestation and lactation period, finding an increased prevalence of MS and hypertension when animals reached adulthood [12]. Another study, trying to find a more limited critical window, administered a high salt diet in rats only during gestation and found an increased incidence of hypertension only in female adults [14]. 

In this study, we undertook the task of determining the effect of a high sucrose diet during a very short-term possible critical window (a period of only 16 days; from day 12 to 28 after birth) on the development of hypertension in the offspring when they reach adulthood. A critical window of the pancreas has been described for this period and there are also important changes in plasma glucose and insulin at this stage [15]. Insulin and glucose modify adult arterial contractility [16] and we have previously described changes in arterial contractility during this critical window [17]. 

Although there are a large number of clinical and animal studies that indicate that the maternal consumption of a diet high in fat and other potential nutrients promotes obesity and increased metabolic risk in offspring [42,43], there are few studies on the long-term effects of high carbohydrate exposure during gestation, lactation, and infancy and their implications for long-term cardiometabolic health in offspring [43,44]. There are even less papers linking the effects of high levels of carbohydrate ingestion to hypertension. Although obesity and MS are frequently associated with hypertension, there might be a direct link between high sugar intake and elevated BP. We do not know of any epidemiological evidence showing a correlation of sugary drinks during infancy and acquired high blood pressure later during adulthood. However, a human population situation that might resemble the condition simulated in the present experiments might be the administration of sweetened tea when babies cry asking for food after recently being breast-fed by the mother, in some countries and epidemiological studies linking high carbohydrate ingestion in critical windows to hypertension would be required to elucidate this issue. Even if some hypertension may be caused by the ingestion of sucrose during early life, it is not the underlying factor in most cases of elevated BP, since very high levels of hypertension are found in Western countries, where giving sucrose to babies is not a common practice. Furthermore, some milk substitutes contain sucrose and/or fructose and therefore some humans feed babies fructose during the breastfeeding period when they complement the nutrition with formula. Soy formula (about 20% of the US market) contain sucrose (which is 50% fructose) instead of lactose. 

In this paper, we found that rats receiving a 30% sucrose concentration in drinking water only during the critical window had increased BP when reaching adulthood. The increase in BP was similar to that observed in rats that received sucrose for seven months (LTS) that included the critical window. Additional controls have previously been carried out in our laboratory consisting of: (a) giving sucrose for six months after the possible critical window period, as a result of which there was also an increase in BP but not as pronounced as when the ingestion of sucrose also included the critical window; and (b) giving sucrose for 16 days during a different period of time than the possible critical window (at three months of age), in which BP was similar to the control rats (data not shown). 

In the present study, we studied and compared three possible mechanisms underlying the development of hypertension after short- and long-term exposure to sucrose. The possible mechanisms were: (1) through the effect of insulin, which might increase endothelin-1 concentration and inhibit eNOS [21]; (2) through an elevation of free FA, mainly OA, whose levels have been associated with the activity of eNOS; and (3) through oxidative stress, since uncoupled eNOS and other enzymes generate reactive oxygen species that react with NO, diminishing its bioavailability and therefore vessel relaxation [22].

Moreover, increased susceptibility to hypertension after STS might be caused by epigenetic marks induced by high glucose and insulin. Histone acetylation might regulate eNOS and SOD gene expression and sirtuins, which are NAD^+^ dependent histone acetylases and ADP-ribosiltransferases (which depend on metabolism levels) might participate in this regulation. Thus, sirtuins might determine vasoconstrictor and vasodilator substance production and release in the vessel, and might modify the lipidic profile or alter OS. Sirtuin 1 regulates the expression of eNOS by deacetylation [45]. Sirtuin 3 is related to inflammatory responses and the expression of SOD. These changes might favor the development of hypertension in the adult [46,47]. Although the effects of the targets of epigenetic control upon obesity and MS have been extensively studied, the targets of hypertension remain to be elucidated. Studying sirtuin expression during STS might be the aim of future studies. 

### 4.1. Effect of the Short- and Long-Term Exposure to Sucrose on Body Variables 

Although, there are several studies on the effect of changes in diet on the increased incidence of MS when the individual reaches adulthood, we did not find that an increased uptake of sucrose during STS elevated the incidence of MS during adulthood. MS with increased weight, abdominal fat, insulin and triglycerides was only found in LTS rats receiving sucrose for seven months. Rats receiving sucrose during STS showed similar body weight, abdominal fat, glucose, insulin and triglycerides as control rats. The lack of effect upon the development of MS might be due to the short term of administration of sucrose used in this study, which was shorter than the period of administration used in other papers. 

It is important to point out that the effect of sucrose during the possible critical window studied includes exposure to this carbohydrate from two sources, namely the milk, since the critical window encompasses the pre-weaning period when the rat pups were still suckling but also began to drink water, and a period in which they only drank the water provided in the cage. There exists little knowledge on how the dietary protein and dietary carbohydrate ratio regulates the expression of metabolic genes in the mother, influencing milk composition. Previous studies have shown that higher breast milk glucose concentrations were associated with greater adiposity in infants [48]. Maternal dietary protein quality, protein quantity and feeding levels have been found to determine mammary protein synthesis and pup weight [49]. The effect of intestinal glucose supply on the mammary utilization of amino acids has been studied in lactating dairy cows [50]. The mammary uptake of all essential amino acids, except arginine and valine, increased linearly with when the supply of glucose was elevated, and the ratio of blood amino acid uptake to milk protein output increased significantly for histidine, methionine and leucine [50]. The duodenal infusion of glucose on carbohydrate metabolism and milk yield in Holstein dairy cows has also been reported [51]. The effect of maternal diet on protein synthesis in isolated mammary acini from lactating rats when low protein diets are administered has also been reported, showing that the rate of synthesis and secretion of alpha-lactalbumin decreased without there being an alteration of the synthesis and secretion of the caseins [52]. In another study, the effect of consuming different percentages of dietary protein and carbohydrates on the expression of genes involved in lipogenesis and protein synthesis in the liver, adipose tissue and the mammary gland were studied, finding a priority for milk synthesis in the mammary gland. The adaptations in the liver and adipose tissue seemed to be responsible for providing nutrients to the mammary gland to sustain milk synthesis [53]. We placed 28-day-old control and STS pups in metabolic cages to determine sucrose ingestion, finding that the STS pups ate and drank less but consumed more daily kcal equivalents. The determination of the importance of exposure to carbohydrates from the two different sources during the critical window around weaning could constitute the aim of future studies.

### 4.2. Nitric Oxide and Endothelin-1 Levels and Expression of eNOS. Regulation by Insulin

We determined the expression of aortic eNOS, which was significantly reduced in rats receiving STS and LTS. There was a tendency for the eNOS expression to be lower in STS than in LTS rats; nevertheless, this difference was not statistically significant. The importance of regulating factors of eNOS expression, such as elevated levels of circulating insulin and insulin resistance (HOMA-IR index) is possibly different in both conditions, since insulin levels and HOMA-IR were increased in LTS rats but not in STS rats. Decreased insulin sensitivity and insulin resistance had already been reported in the MS rats that received the same treatment as our LTS group by using the euglycemic clamp technique [54]. The severity of the alterations in insulin sensitivity in LTS might trigger compensatory mechanisms that are not initiated in a less severe alteration such as STS. 

Our results show that the expression of eNOS was not directly related to the insulin concentration in STS rats, although in our results, these rats showed a non-significant tendency to have increased insulin levels. Therefore, there might be other mechanisms involved. eNOS expression is also regulated by transcription factors such as peroxisome proliferator-activated receptor gamma coactivator 1-α (PCG-1α) and peroxisome proliferator-activated receptor α (PPAR α), which are not evaluated in this paper [55]. eNOS expression could possibly be epigenetically regulated by Sirtuin 1, the expression of which might be determined by high glucose and insulin levels since early life [56].

The seric levels of NO were not in agreement with changes in BP and eNOS expression. This might be due to the fact that the plasma systemic levels that were measured in this study do not always reflect the levels of this mediator released from the endothelium to the media of the aorta. The lack of a NO measurement directly in the aorta is a limitation of this study. Furthermore, NO might be increased by an enhanced expression of inducible nitric oxide synthase (iNOS) [57]. The lack of experimental evidence on this issue is another limitation of this study and should be addressed in future studies.

There are reports in the literature that insulin causes the activation of two separate pathways involved in vasoreactivity: phosphatidyl inositol 3 phosphate (PI3K)/protein kinase B (PKB or AKT)/eNOS pathway and mitogen-activated protein kinase (MAPK)/extracellular signal-regulated kinase (ERK) pathways. PKB phosphorylates eNOS, increasing NO production and vasodilation, while the MAPK pathway results in endothelin-1 production and vasoconstriction [58]. We did not observe the effect of the activation of the PI3K/PKB/eNOS pathway in LTS or STS rats [59]. This might be due to insulin resistance, particularly in LTS rats. A prolonged HOMA-IR increase might be acting as a compensatory mechanism. However, an effect on endothelin-1 probably due to the activation of the MAPK/ERK pathway by insulin might be present in LTS rats [58]. We had previously reported that insulin increases aortic contraction through changes in endothelin-1 release and that these effects are mediated through the ETA and ETB receptors [16]. The activation of this pathway was present in LTS rats that had elevated insulin levels and increased endothelin-1 levels. To our knowledge, this is the first report on the activation of this pathway in the regulation of hypertension in MS. This pathway was not present for STS, which had only a tendency to increased insulin levels in plasma. 

### 4.3. Lipidic Profile, Effect of Free Fatty Acids, Mainly Oleic Acid Diminishing eNOS Activity

Alterations in the lipidic profile are important determinants of HOMA-IR and regulate vascular reactivity, participating in the development of hypertension. The role of triglycerides in hypertension has been previously addressed and an increase of triglycerides in MS has been previously reported [60,61]. The triglycerides were not increased in the STS rats. Therefore, triglycerides play a role in LTS-induced hypertension, but not in STS-induced hypertension.

HDL may stimulate phosphorylated eNOS, since it has been described that endothelial cells incubated with HDL exhibit an increase in eNOS activity [62]. The HDL levels were increased in LTS rats but not in STS rats.

High levels of non-esterified FA and of monounsaturated FA have also been proposed as contributors to HOMA-IR and hypertension [19,60,61]. The biosynthesis of the monounsaturated and polyunsaturated FA results from the activity of desaturases [63]. These enzymes contribute to the control of membrane FA-dependent structure disorders [64]. Alterations in MUFA and serum FA can be reflected in changes in endothelial cell membrane composition [65]. These changes may interfere with the accessibility to receptors and may modify membrane ionic transport and enzymatic activities [66]. 

As in previous papers on the MS model [19,60,61], we found altered levels of non-esterified FA and of monounsaturated FA, which have been proposed as contributors to insulin resistance and hypertension. These changes were not observed in STS rats. Changes in the FA composition of several tissues can be attributed to: (a) the type of diet ingested; (b) alterations in fatty acid metabolism, including their oxidation; (c) incorporation in the phospholipids of the membrane; or (d) to the desaturase activities [19,67].

FA are important components of the phospholipids in the lipid bilayer of the cell membrane. They have an important regulatory function, as precursors of prostaglandins, thromboxanes and leukotrienes [68]. Abnormalities in FA metabolism are important in the pathogenesis of the endothelial cell membrane dysfunction and can be associated with alterations in vascular reactivity [69]. FA can stimulate NO production by activating NF-kB and increasing the expression and activity of iNOS [70]. Oleic acid modulates numerous functions such as cytokine release, apoptosis, necrosis and OS [71]. It also plays an important role in the pathogenesis of endothelial dysfunction and atherosclerosis [57]. OA stimulates iNOS and may lower phosphorylated eNOS expression. It may also increase NF-kB, which may in turn raise the level of expression of iNOS and consequently cause a NO overproduction [57]. These changes could result in the tendency to an increased level of NO found in STS rats, without reaching statistical significance. 

Arachidonic acid is recognized as a second messenger that can affect cell function by modulating intracellular signal transduction [72]. In cultures of human cells, arachidonic acid induces a significant increase in iNOS gene expression [72]. Arachidonic acid was only decreased in LTS rats. Therefore, the changes in the lipidic profile seem to participate in some characteristics of the MS developed in LTS rats, but not in the development of hypertension. The lipidic profile was not altered in the STS rats and consequently in the development of hypertension in this model. 

### 4.4. Effect of Oxidative Stress Diminishing the Bioavailability of NO

An imbalance between the production of reactive oxygen species (ROS) and the antioxidant capacity of the biological system results in OS. OS alters the essential processes, possibly becoming the origin of tissue damage in the organism, and requires the rapid detoxification of intermediate reactants or of the repair of the damage it causes [73,74]. OS and vascular dysfunction have been associated with alterations of the contractile function and endothelium-mediated relaxation [75,76]. OS is involved in cardiovascular diseases, including hypertension, arrhythmias, coronary arterial disease, left ventricular hypertrophy, aortic dilatation, aortic dissection, and congestive heart failure [64,77,78]. ROS and reactive nitrogen species (RNS) are produced in these diseases through different pathways such as mitochondrial xanthine oxidase and NADPH oxidase (Nox). eNOS also plays a relevant role in these diseases [79,80,81,82,83,84,85,86]. Additionally, it has been reported that ROS and LPO lead to DNA oxidative damage and to high levels of 8-iso-prostaglandin F2α (8-iso-PGF2α) in patients with essential hypertension [87]. Our results show that the total antioxidant capacity was decreased in STS and LTS, and might contribute as a possible cause of hypertension. However, there were no changes in LPO as a result of the short- or long-term exposure to sucrose. These results are in line with previous results that show that LPO is not significantly elevated in MS rats, although the total antioxidant capacity was reduced [88]. Organisms have evolved multiple defense lines to prevent oxidative damage, ranging from antioxidant enzymes to low molecular weight antioxidants, and also specific cellular components that repair oxidatively damaged molecules [89]. Perhaps in our STS model and in this series of LTS rats, the components that repair oxidatively-damaged molecules are enhanced and lipoperoxidation was therefore not observed. The lack of lipoperoxidation might also be explained by the antioxidant action of NO, which reacts with free radicals, constituting a potent inhibitor of lipoperoxidation propagation. NO is even able to diminish LDL oxidation, diminishing atherosclerotic lesion formation [90,91,92,93]. Preliminary results from our group indicate that there is lipoperoxidation at the end of the critical window (on postnatal day 28, data not shown) that does not persist to adulthood. However, this condition might lead to epigenetic cues that could persist until the animals reach the adult stage and participate in the regulation of the expression of the genes participating in BP regulation.

SOD isoforms are important within the vascular wall in normal conditions and in diseased states in humans. Cu-Zn-SOD expression is relatively high in all cell types, including blood cells, and it accounts for 50% to 80% of the total activity, being, therefore, the predominant isoform. SOD2 or Mn-SOD is responsible for 2% of the activity and the remaining 12% of the activity may be due to extracellular SOD [94]. In this study we analyzed the protein expression of SOD1 and SOD2. SOD1 expression was not modified in LTS or STS rats, while SOD2 expression was decreased in both experimental groups. The study of the activity of these enzymes might also be modified and it might constitute the aim of a future study. Since the mitochondria is the main source of free radicals and there are several reports in the literature that state that SOD2 deficiency is related to mitochondrial oxidative stress and to atherosclerosis [95], we suggest that deficiency in this SOD isoform plays an important role in the vascular dysfunction found in LTS rats. 

Although the SOD2 present in the mitochondria accounts for only a small percentage of the total SOD activity, it plays an important role in the crosstalk of this organelle with the nucleus, participating in the establishment of epigenetic marks and the programming of diseases from early life [96]. The beneficial function of SOD as an antioxidant in different illnesses [97], both in animal models and in patients with or without an active inflammatory disease, has been described [98]. We found no changes in SOD1 in the short or long- term exposure to sucrose; however, SOD2 was decreased in both conditions and could participate in the underlying causes of sucrose-induced hypertension. SOD has been described as being under epigenetic control by Sirtuin 3 [99]. In conclusion, the reduced total antioxidant capacity and expression of SOD2 might participate as part of the mechanisms that underlie hypertension in both models.

## 5. Conclusions

In conclusion high sucrose intake during the STS increases arterial BP when individuals reach adulthood. Therefore, there is a postnatal critical window in vascular development that induces increased susceptibility to hypertension and that takes place simultaneously to the critical window of the pancreas. The mechanism underlying elevated BP in these rats involved a decrease in eNOS expression, and increased oxidative stress reflected in a decreased total antioxidant capacity and a reduced expression of SOD2.

In LTS there was also hypertension that reached a similar level as in STS rats. The increase in BP was accompanied by increased insulin levels, insulin resistance, and increased levels of endothelin-1. There were also alterations in the lipid profile that were not correlated to NO levels or eNOS expression. SOD2 expression and the antioxidant capacity were decreased in these rats. Therefore, although there is hypertension and decreased expression of eNOS in both models, the mechanisms underlying the regulation of the expression of this enzyme seem to be different. The mechanism involved in both situations have similarities (participation of eNOS and SOD expression and OS) and differences (elevated endothelin-1 levels increased only in LTS, fatty acids and arachidonic acid only participate in LTS). Finally, it is important to control diet during the early stages of development to reduce the risk of developing hypertension when reaching adulthood. 

## Figures and Tables

**Table 1:** The effects of long-term (LTS) and short-term (STS) administration of sucrose on body characteristics and biochemical parameters. Data represent mean ± standard error of the mean (SEM), # *p* < 0.01, after one-way analysis of variance (ANOVA), *n* = 10 determinations from 10 different rats from each group.

	Control	LTS	STS
Weight (g)	509.20 ± 3.68	419.67 ± 5.24	496.00 ± 5.00
Visceral adipose tissue (g)	4.08 ± 0.44	9.82 ± 1.16 #	4.23 ± 0.41
Systolic pressure (mm Hg)	105.4 ± 0.75	125.81 ± 1.13 #	125.47 ± 1.07 #
Glucose (mg/dL)	110.63 ± 7.6	100.73 ± 5.61	93.36 ± 3.5
Insulin (µU/mL)	7.61 ± 0.73	13.62 ± 1.18 #	9.66 ± 0.52
HOMA-IR	1.36 ± 0.16	2.48 ± 0.29 #	2.03 ± 0.17

**Table 2:** The effects of long-term (LTS) and short- term (STS) administration of sucrose on serum triglycerides, total cholesterol (TC), high- density lipoprotein cholesterol (HDL-C), and non-HDL-C levels. Data represent mean ± SEM, # *p* < 0.01 after one-way ANOVA, *n* = 10 determinations from 10 different rats from each group.

	Control	LTS	STS
Triglycerides (mg/dL)	66.48 ± 9.55	126.95 ± 8.10 #	47.29 ± 4.98
Total cholesterol (mg/dL)	51.34 ± 4.11	54.77 ± 4.97	62.40 ± 3.94
Cholesterol-HDL (mg/dL)	26.32 ± 1.96	19.76 ± 1.65	34.28 ± 2.6
Cholesterol non-HDL (mg/dL)	23.54 ± 1.60	31.59 ± 3.72	26.25 ± 1.92

**Table 3:** The effects of long-term (LTS) and short-term (STS) administration of sucrose on seric fatty acid composition. Data represent mean ± SEM, # *p* < 0.01, *n* = 8 determinations from 8 different rats from each group.

Fatty Acid (%)	Control	LTS	STS
Palmitic	27.21 ± 0.67	33.53 ± 0.57	27.22 ± 0.62
Stearic	32.98 ± 1.03	20.48 ± 1.40 #	33.15 ± 0.75
Oleic	24.94 ± 0.96	20.93 ± 1.52	24.24 ± 0.42
Linoleic	2.47 ± 0.29	10.46 ± 1.20 #	5.29 ± 0.59
Arachidonic	11.27 ± 0.90	8.33 ± 0.82 #	10.20 ± 0.53

## Abbreviations

ANOVA	analysis of variance
BP	blood pressure
CZE	capillary zone electrophoresis
ERK	extracellular signal–regulated kinases
eNOS	endothelial nitric oxide synthase
ETA	endothelin-1 receptor A
ETb	endothelin-1 receptor B
FA	fatty acids
HDL	high-density lipoproteins
HOMA-IR	homeostasis model assessment of insulin resistance
IDL	intermediate-density lipoproteins
iNOS	inducible nitric oxide synthase
LDL	low-density lipoproteins
LPO	lipoperoxidation
LTS	long-term sucrose
MAPK	mitogen activated protein kinase
MS	metabolic syndrome
MUFA	monounsaturated fatty acids
NF-kB	necrosis factor kB
NAD+	nicotinamide adenine dinucleotide
NO	nitric oxide
OA	oleic acid
OS	oxidative stress
PI3K	phosphatidyl inositol 3 phosphate
PKB, AKT	protein kinase B
PCG-1α	peroxisome proliferator-activated receptor gamma coactivator 1-α
PPAR α	peroxisome proliferator-activated receptor α
PUFA	polyunsaturated fatty acids
RIA	radioimmunoassay
SEM	standard errors of the mean
SOD1	superoxide dismutase 1; Cu-Zn superoxide dismutase
SOD2	superoxide dismutase 2; Mn superoxide dismutase
STS	short-term sucrose
TC	total cholesterol
TGs	triglycerides
VLDL	very-low-density lipoproteins

## References

[1] Lucas A. (1998). Programming by early nutrition. An experimental approach. J. Nutr..

[2] Girard J. (1990). Metabolic adaptations to change of nutrition at birth. Biol. Neonate.

[3] Ozanne S.E., Hales C.N. (2002). Early programming of glucosa-insulin metabolism. Trends Endocrinol. Metab..

[4] Aalinkeel R., Srinivasan M., Kalhan S.C., Laychock S.G., Patel M.S. (1999). A dietary intervention (high carbohydrate) during the neonatal period causes islet dysfunction in rats. Am. J. Physiol..

[5] Song F., Srinivasan M., Aalinkeel R., Patel M.S. (2001). Use of a cDNA array for the identification of genes induced in islets of suckling rats by a high-carbohydrate nutritional intervention. Diabetes.

[6] Srinivasan M., Aalinkeel R., Song F., Mitrani P., Pandya J.D., Strutt B., Hill D.J., Patel M.S. (2006). Maternal hyperinsulinemia predisposes rat fetuses for hyperinsulinemia, and adult-onset obesity and maternal mild food restriction reverses this phenotype. Am. J. Physiol. Endocrinol. Metab..

[7] Lawlor D.A., Smith G.D. (2005). Early life determinants of adult blood pressure. Curr. Opin. Nephrol. Hypertens..

[8] Langley S.C., Jackson A.A. (1994). Increased systolic blood pressure in adult rats induced by fetal exposure to maternal low-protein diets. Clin. Sci..

[9] Woods L.L., Weeks D.L., Rasch R. (2004). Programming of adult blood pressure by maternal protein restriction: Role of nephrogenesis. Kidney Int..

[10] Alexander B.T. (2003). Placental insufficiency leads to development of hypertension in growth-restricted offspring. Hypertension.

[11] Schreuder M.F., van Wijk J.A.E., Delemarre-van de Waal H.A. (2006). Intrauterine growth restriction increases blood pressure and central pulse pressure measured with telemetry in aging rats. J. Hypertens..

[12] Samuelsson A.M., Matthews P.A., Jansen E., Taylor P.D., Poston L. (2013). Sucrose feeding in mouse pregnancy leads to hypertension, and sex-linked obesity and insulin resistance in female offspring. Front. Physiol..

[13] Contreras R.J., Wong D.L., Henderson R., Curtis K.S., Smith J.C. (2000). High dietary NaCl early in development enhances mean arterial pressure of adult rats. Physiol. Behav..

[14] Porter J.P., King S.H., Honeycut A.D. (2007). Prenatal high-salt diet in the Sprague-Dawley rat programs blood pressure and heart rate hyperresponsiveness to stress in adult female offspring. Am. J. Physiol. Regul. Integr. Comp. Physiol..

[15] Aguayo-Mazzucato C., Sanchez-Soto C., Godinez-Puig V., Gutierrez-Ospina G., Hiriart M. (2006). Restructuring of pancreatic islets and insulin secretion in a postnatal critical window. PLoS ONE.

[16] Nava P., Collados M.T., Massó F., Guarner V. (1997). Endothelin-1 mediation of insulin and glucose induced changes in vascular contractility. Hypertension.

[17] Rubio-Ruiz M.E., Vargas-Gonzalez A., Monter-Garrido M., Guarner-Lans V. (2010). Aortic vaso-reactivity during a postnatal critical window of the pancreas in rats. Heart Vessel..

[18] Wallace D.C. (2005). A mitochondrial paradigm of metabolic and degenerative diseases, aging, and cancer: A dawn for evolutionary medicine. Annu. Rev. Genet..

[19] Wallace D.C., Fan W. (2010). Energetics, epigenetics, mitochondrial genetics. Mitochondrion.

[20] Shaughnessy D.T., McAllister K., Worth L., Haugen A.C., Meyer J.N., Domann F.E., Van Houten B., Mostoslavsky R., Bultman S.J., Baccarelli A.A. (2014). Mitochondria, energetics, epigenetics, and cellular responses to stress. Environ. Health Perspect..

[21] Hernández-Díaz A., Arana-Martínez J.C., Carbó R., Espinosa-Cervantes R., Sánchez-Muñoz F. (2016). Omentin: Role in insulin resistance, inflammation and cardiovascular protection. Arch. Cardiol. Mex..

[22] Pérez-Torres I., Zúñiga Muñoz A., Beltrán-Rodríguez U., Díaz-Díaz E., Martínez-Memije R., Guarner Lans V. (2014). Modification of the liver fatty acids by Hibiscus sabdariffa Linnaeus (Malvaceae) infusion, its possible effect on vascular reactivity in a metabolic syndrome model. Clin. Exp. Hypertens..

[23] Baños G., Carvajal K., Cardoso G., Zamora J., Franco M. (1997). Vascular reactivity and effect of serum in a rat model of hypertriglyceridemia and hypertension. Am. J. Hypertens..

[24] Bradford M.M. (1976). A rapid and sensitive method for the quantitation of microgram quantities of protein utilizing the principle of protein-dye binding. Anal. Biochem..

[25] Rubio-Ruíz M.E., Del Valle-Mondragón L., Castrejón-Tellez V., Carreón-Torres E., Díaz-Díaz E., Guarner-Lans V. (2014). Angiotensin II and 1–7 during aging in metabolic syndrome rats. Expression of AT1, AT2 and Mas receptors in abdominal white adipose tissue. Peptides.

[26] Pérez Méndez O., García Hernández L. (2007). High-Density Lipoproteins (HDL) size and composition are modified in the rat by a diet supplemented with ‘Hass’ avocado (Persea americana Miller). Arch. Cardiol. Mex..

[27] Akondi R.N., Trexler R.V., Pfiffner S.M., Mouser P.J., Sharma S. (2017). Modified Lipid Extraction Methods for Deep Subsurface Shale. Front. Microbiol..

[28] Pérez-Torres I., Roque P., El Hafidi M., Diaz-Diaz E., Baños G. (2009). Association of renal damage and oxidative stress in a rat model of metabolic syndrome. Influence of gender. Free Radic. Res..

[29] Benzie I.F., Strain J.J. (1996). The ferric reducing ability of plasma (FRAP) as a measure of “antioxidant power”: The FRAP assay. Anal. Biochem..

[30] Tenorio F.A., del Valle L., Gustavo Pastelín H. (2005). Validación de un método analítico espectrofotométrico para la cuantificación de metabolitos estables de óxido nítrico en fluidos biológicos. Rev. Mex. Cienc. Farm..

[31] Kumarathasan P., Goegan P., Vincent R. (2001). An automated high-performance liquid chromatography fluorescence method for the analyses of endothelin-1s in plasma samples. Anal. Biochem..

[32] Raitakari O.T., Juonala M., Viikari J.S. (2005). Obesity in childhood and vascular changes in adulthood: Insights into the Cardiovascular Risk in Young Finns Study. Int. J. Obes..

[33] Li L., Law C., Power C. (2007). Body mass index throughout the life-course and blood pressure in mid-adult life: A birth cohort study. J. Hypertens..

[34] Hardy R., Wadsworth M.E., Langenberg C., Kuh D. (2004). Birthweight, childhood growth, and blood pressure at 43 years in a British birth cohort. Int. J. Epidemiol..

[35] Mu J.J., Liu Z.Q., Yang J., Ren J., Liu W.M., Xu X.L., Xiong S.E. (2008). Association between higher blood pressure level in children and adult blood pressure: 17 years follow-up results. Zhonghua Xin Xue Guan Bing Za Zhi.

[36] Osmond C., Barker D.J. (2000). Fetal, infant, and childhood growth are predictors of coronary heart disease, diabetes, and hypertension in adult men and women. Environ. Health Perspect..

[37] Berenson G.S., Srinivasan S.R., Hunter S.M., Nicklas T.A., Freedman D.S., Shear C.L., Webber L.S. (1989). Risk factors in early life as predictors of adult heart disease: The Bogalusa Heart Study. Am. J. Med. Sci..

[38] Grobbee D.E., van Hooft I.M., de Man S.A. (1990). Determinants of blood pressure in the first decades of life. J. Cardiovasc. Pharmacol..

[39] Seki Y., Williams L., Vuguin P.M., Charron M.J. (2012). Minireview: Epigenetic programming of diabetes and obesity: Animal models. Endocrinology.

[40] Chandrasekera P.C., Pippin J.J. (2014). Of Rodents and Men: Species-Specific Glucose Regulation and Type 2 Diabetes Research. Altex.

[41] Ventura E.E., Davis J.N., Goran M.I. (2011). Sugar content of popular sweetened beverages based on objective laboratory analysis: Focus on fructose content. Obesity.

[42] Xavier R., Sreeramanan S., Diwakar A., Sivagnanam G., Sethuraman K.R. (2007). Soft Drinks and Hard Facts: A Health Perspective. ASEAN Food J..

[43] Goran M.I., Martin A.A., Alderete T.L., Fujiwara H., Fields D.A. (2017). Fructose in Breast Milk Is Positively Associated with Infant Body Composition at 6 Months of Age. Nutrients.

[44] Zheng J., Feng Q., Zhang Q., Wang T., Xiao X. (2016). Early Life Fructose Exposure and Its Implications for Long-Term Cardiometabolic Health in Offspring. Nutrients.

[45] Mattagajasingh I., Kim C.S., Naqvi A., Yamamori T., Hoffman T.A., Jung S.B., DeRicco J., Kasuno K., Irani K. (2007). SIRT1 promotes endothelium-dependent vascular relaxation by activating endothelial nitric oxide synthase. PNAS.

[46] Turgeon P.J., Sukumar A.N., Marsden P.A. (2014). Epigenetics of Cardiovascular Disease–A New “Beat” in Coronary Artery Disease. Med. Epigenet..

[47] Farghali H., Kutinová Canoová N., Lekic N. (2013). Resveratrol and Related Compounds as Antioxidants with an Allosteric Mechanism of Action in Epigenetic Drug Targets. Physiol. Res..

[48] Plagemann A., Harder T., Franke K., Kohlhoff R. (2002). Long-term impact of neonatal breast-feeding on body weight and glucose tolerance in children of diabetic mothers. Diabetes Care.

[49] Sampson D.A., Hunsaker H.A., Jansen G.R. (1986). Dietary protein quality, protein quantity and food intake: Effects on lactation and on protein synthesis and tissue composition in mammary tissue and liver in rats. J. Nutr..

[50] Rulquin H., Rigout S., Lemosquet S., Bach A. (2004). Infusion of glucose directs circulating amino acids to the mammary gland in well-fed dairy cows. J. Dairy Sci..

[51] Lemosquet S., Delamaire E., Lapierre H., Blum J.W., Peyraud J.L. (2009). Effects of glucose, propionic acid, and nonessential amino acids on glucose metabolism and milk yield in Holstein dairy cows. J. Dairy Sci..

[52] Geursen A., Carne A., Grigor M.R. (1987). Protein synthesis in mammary acini isolated from lactating rats: Effect of maternal diet. J. Nutr..

[53] Velázquez-Villegas L.A., Tovar A.R., López-Barradas A.M., Torres N. (2013). The dietary protein/carbohydrate ratio differentially modifies lipogenesis and protein synthesis in the mammary gland, liver and adipose tissue during gestation and lactation. PLoS ONE.

[54] El-Hafidi M., Franco M., Ruiz Ramírez A., Sosa J.S., Flores J.A.P., Acosta O.L., Salgado M.C., Cardoso-Saldaña G. (2018). Glycine Increases Insulin Sensitivity and Glutathione Biosynthesis and Protects against Oxidative Stress in a Model of Sucrose-Induced Insulin Resistance. Oxid. Med. Cell. Longev..

[55] Craige S.M., Kröller-Schön S., Li C., Kant S., Cai S., Chen K., Contractor M.M., Pei Y., Schulz E., Keaney J.F. (2016). PGC-1α dictates endothelial function through regulation of eNOS expression. Sci. Rep..

[56] De Nigris V., Pujadas G., La Sala L., Testa R., Genovese S., Ceriello A. (2015). Short-term high glucose exposure impairs insulin signaling in endothelial cells. Cardiovasc. Diabetol..

[57] Han F., Guo Y., Xu L., Hou N., Han F., Sun X. (2015). Induction of haemeoxygenase-1 Directly Improves Endothelial Function in Isolated Aortas from Obese Rats through the Ampk-Pi3k/Akt-Enos Pathway. Cell. Physiol. Biochem..

[58] El Hafidi M., Valdez R., Baños G. (2000). Possible relationship between altered fatty acid composition of serum, platelets, and aorta and hypertension induced by sugar feeding in rats. Clin. Exp. Hypertens..

[59] Ruiz-Ramírez M.A., Chávez-Salgado J., Peñeda-Flores A., Zapata E., Masso F., El-Hafidi M. (2011). High-sucrose diet increases ROS generation, FFA accumulation, UCP2 level, and proton leak in liver mitochondria. Am. J. Physiol. Endocrinol. Metab..

[60] Drew B.G., Fidge N.H., Gallon-Beaumier G., Kemp B.E., Kingwell B.A. (2004). High-density lipoprotein and apolipoprotein AI increase endothelial NO synthase activity by protein association and multisite phosphorylation. Proc. Natl. Acad. Sci. USA.

[61] Priante G., Musacchio E., Pagnin E., Calò L.A., Baggio B. (2005). Specific effect to arachidonic acid on inducible nitric oxide synthase mRNA expression in human osteoblastic cells. Clin. Sci..

[62] Verlengia R., Gorjão R., Kanunfre C.C., Bordin S., de Lima T.M., Newsholme P., Curi R. (2003). Genes regulated by arachidonicand oleic acids in Raji cells. Lipids.

[63] Rupp H., Turcani M., Ohkubo T., Maisch B., Brilla C.G. (1996). Dietary linolenic acid mediated increasein vascular prostacyclin formation. Mol. Cell. Biochem..

[64] Das U.N. (2010). A defect in D6 and D5 desaturases may be a factor in the initiation and progression of insulin resistance, the metabolic syndrome and ischemic heart disease in South Asians. Lipids Health Dis..

[65] Garaulet M., Hernandez-Morante J.J., Tebar F.J., Zamora S. (2011). Relation between degree of obesity and site-specific adipose tissue fatty acid composition in a Mediterranean population. Nutrition.

[66] Vila L. (2004). Cyclooxygenase and 5-lipoxygenase pathways in the vessel wall: Role in atherosclerosis. Med. Res. Rev..

[67] Høstmark A.T., Haug A. (2013). Percentages of oleic acid and arachidonic acid are inversely related in phospholipids of human sera. Lipids Health Dis..

[68] De Lima T.M., de Sa Lima L., Scavone C., Curi R. (2006). Fatty acid control of nitric oxide production by macrophages. FEBS Lett..

[69] Cury-Boaventura M.F., Gorjão R., de Lima T.M., Newsholme P., Curi R. (2006). Comparative toxicity of oleic and linoleic acid on human lymphocytes. Life Sci..

[70] Park J.Y., Kim Y.M., Song H.S., Park K.Y., Kim Y.M., Kim M.S., Pak Y.K., Lee I.K., Lee J.D., Park S.J., Lee K.U. (2003). Oleic acid induces endothelin-1-1 expression through activation of protein kinase C and NF-kappa B. Biochem. Biophys. Res. Commun..

[71] Di Marzo V. (1995). Arachidonic acid and eicosanoids as targets and effectors in second messenger interactions. Prostaglandins Leukot. Essent. Fat. Acids.

[72] Xia Y., Tsai A.L., Berka V., Zweier J.L. (1998). Superoxide generation from endothelial nitric-oxide synthase: A Ca2+/calmodulin dependent and tetrahydrobiopterin regulatory process. J. Biol. Chem..

[73] Sawyer D.B., Siwik D.A., Xiao L., Pimentel D.R., Singh K., Colucci W.S. (2002). Role of oxidative stress in myocardial hypertrophy and failure. J. Mol. Cell. Cardiol..

[74] Kane M.O., Etienne-Selloum N., Madeira S.V.F., Sarr M., Walter A., Dal-Ros S., Schott C., Chataigneau T., Schini-Kerth V.B. (2010). Endothelium-derived contracting factors mediate the Ang II induced endothelial dysfunction in the rat aorta: Preventive effect of red wine polyphenols. Pflugers Arch. Eur. J. Physiol..

[75] Serpillon S., Floyd B.C., Gupte R.S., George S., Kozicky M., Neito V., Recchia F., Stanley W., Wolin M.S., Gupte S.A. (2009). Superoxide production by NAD(P)H oxidase and mitochondria is increased in genetically obese and hyperglycemic rat heart and aorta before the development of cardiac dysfunction. The role of glucose- 6-phosphate dehydrogenase-derived NADPH. Am. J. Physiol. Heart Circ. Physiol..

[76] Bolli R., Jeroudi M.O., Patel B.S., Aruoma O.I., Halliwell B., Lai E.K., McCay P.B. (1989). Marked reduction of free radical generation and contractile dysfunction by antioxidant therapy begun at the time of reperfusion: Evidence that myocardial ‘stunning’ is a manifestation of reperfusion injury. Circ. Res..

[77] Charniot J.C., Bonnefont-Rousselot D., Albertini J.P., Zerhouni K., Dever S., Richard I., Nataf P., Pavie A., Monsuez J.J., Delattre J., Artigou J.E. (2007). Oxidative stress implication in a new ex-vivo cardiac concordant xenotransplantation model. Free Radic. Res..

[78] Griendling K.K., Sorescu D., Ushio-Fukai M. (2000). NAD(P)H oxidase: Role in cardiovascular biology and disease. Circ. Res..

[79] Griendling K.K., Sorescu D., Lasségue B., Ushio-Fukai M. (2000). Modulation of protein kinase activity and gene expression by reactive oxygen species and their role in vascular physiology and pathophysiology. Arterioscler. Thromb. Vasc. Biol..

[80] Murdoch C.E., Zhang M., Cave A.C., Shah A.M. (2006). NADPH oxidase-dependent redox signalling in cardiac hypertrophy, remodelling and failure. Cardiovasc. Res..

[81] Minhas K.M., Saraiva R.M., Schuleri K.H., Lehrke S., Zheng M., Saliaris A.P., Berry C.E., Vandegaer K.M., Li D., Hare J.M. (2006). Xanthine oxidoreductase inhibition causes reverse remodeling in rats with dilated cardiomyopathy. Circ. Res..

[82] Ide T., Tsutsui H., Kinugawa S., Utsumi H., Kang D., Hattori N., Uchida K., Arimura K.I., Egashira K., Takeshita A. (1999). Mitochondrial electron transport complex I is a potential source of oxygen free radicals in the failing myocardium. Circ. Res..

[83] Umar S., Van Der Laarse A. (2010). Nitric oxide and nitric oxide synthase isoforms in the normal, hypertrophic, and failing heart. Mol. Cell. Biochem..

[84] Takimoto E., Champion H.C., Li M., Ren S., Rodriguez E.R., Tavazzi B., Lazzarino G., Paolocci N., Gabrielson K.L., Wang Y., Kass D.A. (2005). Oxidant stress from nitric oxide synthase-3 uncoupling stimulates cardiac pathologic remodeling from chronic pressure load. J. Clin. Investig..

[85] Saavedra W.F., Paolocci N., St. John M.E., Skaf M.W., Stewart G.C., Xie J.S., Harrison R.W., Zeichner J., Mudrick D., Marbán E. (2002). Imbalance between xanthine oxidase and nitric oxide synthase signaling pathways underlies mechano energetic uncoupling in the failing heart. Circ. Res..

[86] White C.N., Liu C.C., Garcia A., Hamilton E.J., Chia K.K., Figtree G.A., Rasmussen H.H. (2010). Activation of cAMP dependent signaling induces oxidative modification of the cardiac Na+-K+ pump and inhibits its activity. J. Biol. Chem..

[87] Belik J., Jankov R.P., Pan J., Yi M., Pace-Asciak C.R., Tanswell A.K. (2003). Effect of 8-isoprostaglandin F2α on the newborn rat pulmonary arterialmuscle and endothelium. J. Appl. Physiol..

[88] Guerra R.C., Zúñiga-Muñoz A., Guarner-Lans V., Díaz-Díaz E., Tena-Betancourt C.A., Pérez-Torres I. (2014). Modulation of the activities of cabalase, Cu-Zn, Mn Superoxide dismutase and glutathione peroxidase in adipocytes from ovariectomized female rats with metabolic syndrome. Int. J. Endocrinol..

[89] Constantini D., Verhulst S. (2009). Does high antioxidant capacity indicate low oxidative stress?. Funct. Ecol..

[90] Rubbo H., Parthasarathy S., Barnes S., Kirk M., Kalyanaraman B., Freeman B.A. (1995). Nitric oxide inhibition of lipoxygenase-dependent liposome and low- density lipoprotein oxidation: Termination of radical chain propagation reactions and formation of nitrogen-containing oxidized lipid derivatives. Arch. Biochem. Biophys..

[91] Rubbo H., Freeman B.A. (1996). Nitric oxide regulation of lipid oxidation reactions: Formation and analysis of nitrogen-containing oxidized lipid derivatives. Methods Enzymol..

[92] Rubbo H., Radi R., Anselmi D., Kirk M., Barnes S., Eiserich J., Freeman B.A. (2000). Nitric oxide reaction with lipid peroxyl radicals spares α-tocopherol during lipid peroxidation: Greater oxidant protection from the pair nitric oxide/α-tocopherol than α-tocopherol/ascorbate. J. Biol. Chem..

[93] Trostchansky A., Batthyány C., Botti H., Radi R., Denicola A., Rubbo H. (2001). Formation of lipid-protein adducts in low-density lipoprotein by fluxes of peroxynitrite and its inhibition by nitric oxide. Arch. Biochem. Biophys..

[94] Didion S.P., Ryan M.J., Didion L.A., Fegan P.E., Sigmund C.D., Faraci F.M. (2002). Increased superoxide and vascular dysfunction in CuZnSOD-deficient mice. Circ. Res..

[95] Vendrov A.E., Stevenson M.D., Alahari S., Pan H., Wickline S.A., Madamanchi N.R., Runge M.S. (2017). Attenuated Superoxide Dismutase 2 Activity Induces Atherosclerotic Plaque Instability During Aging in Hyperlipidemic Mice. J. Am. Heart Assoc..

[96] Zapata-Martín del Campo C.M., Martínez-Rosas M., Guarner-Lans V. (2018). Epigenetics of Subcellular Structure Functioning in the Origin of Risk or Resilience to Comorbidity of Neuropsychiatric and Cardiometabolic Disorders. Int. J. Mol. Sci..

[97] Mármol F., Sánchez J., López D., Martinez N., Rosello-Catafau J., Mitjavila M.T., Puig-Parellada P. (2007). Loss of adaptation to oxidative stress as a mechanism for aortic damage in aging rats. J. Physiol. Biochem..

[98] Lubrano V., DiCecco P., Zucchelli G.C. (2006). Role of superoxide dismutase in vascular inflammation and in coronary artery disease. Clin. Exp. Med..

[99] Chen Y., Zhang J., Lin Y., Lei Q., Guan K.L., Zhao S., Xiong Y. (2011). Tumour suppressor SIRT3 deacetylates and activates manganese superoxide dismutase to scavenge ROS. EMBO Rep..

